# Designing C_9_N_10_ Anchored Single Mo Atom as an Efficient Electrocatalyst for Nitrogen Fixation

**DOI:** 10.3390/molecules29194768

**Published:** 2024-10-09

**Authors:** Yibo Chen, Liang Chen, Xinyu Zhang, Pengyue Zhang

**Affiliations:** 1 Intelligent Manufacturing Institute, Hebei Vocational University of Industry and Technology, Shijiazhuang 050091, China; yibochen@yeah.net (Y.C.); chen_liangd@hotmail.com (L.C.); 2 State Key Laboratory of Metastable Materials Science and Technology, Yanshan University, Qinhuangdao 066004, China; xyzhang@ysu.edu.cn

**Keywords:** single-atom catalyst, first-principle calculations, nitrogen reduction reaction, graphitic carbon nitride

## Abstract

Electrochemical nitrogen reduction reaction (NRR) is a promising route for realizing green and sustainable ammonia synthesis under ambient conditions. However, one of the major challenges of currently available Single-atom catalysts (SACs) is poor catalytic activity and low catalytic selectivity, which is far away from the requirements of industrial applications. Herein, first-principle calculations within the density functional theory were performed to evaluate the feasibility of a single Mo atom anchored on a g-C_9_N_10_ monolayer (Mo@g-C_9_N_10_) as NRR electrocatalysts. The results demonstrated that the gas phase N_2_ molecule can be sufficiently activated on Mo@g-C_9_N_10_, and N_2_ reduction dominantly occurs on the active Mo atom via the preferred enzymatic mechanism, with a low limiting potential of −0.48 V. In addition, Mo@g-C_9_N_10_ possesses a good prohibition ability for the competitive hydrogen evolution reaction. More impressively, good electronic conductivity and high electron transport efficiency endow Mo SACs with excellent activity for electrocatalytic N_2_ reduction. This theoretical research not only accelerates the development of NRR electrocatalysts but also increases our insights into optimizing the catalytic performance of SACs.

## 1. Introduction

Ammonia (NH_3_), as an irreplaceable chemical feedstock, has made substantial contributions to the existence and development of current human society [1,2]. It cannot only be heavily applied in the modern industrial synthesis of items such as fertilizers, pesticides, and pharmaceuticals, but also as a new, safe, and ecofriendly energy carrier [3,4,5,6,7]. Nonetheless, the large scale industrial NH_3_ production is primarily based on the conventional Haber–Bosch process, which demands extremely severe reaction conditions (temperature 300~550 °C and pressure 150~350 atm) [8,9] and leads to the huge energy consumption and the substantial greenhouse gas CO_2_ emissions. Hence, it is imperative to develop an energy-saving and environmentally friendly strategy that can be carried out under mild reaction conditions to achieve an efficient conversion of N_2_ to NH_3_ and substitute for traditional thermal catalytic technology. In recent years, electrocatalytic N_2_ reduction employing the H_2_O molecule as the hydrogen source has attracted widespread attention from researchers because of the relatively moderate reaction conditions, high efficiency, and zero emission of greenhouse gases [10]. However, electrocatalytic N_2_ reduction cannot currently meet the practical industrial applications due to the extremely high bonding energy of stable N≡N triple bond as well as the competitive hydrogenation evolution reaction.

Single-atom catalysts (SACs) with the isolated metal atom decorated on a two-dimensional monolayer have become a research frontier in the catalytic field, owing to their unified active center, adjustable coordination environment, and maximum atomic utilization. Previous studies demonstrated that SACs exhibit tremendous potential in various catalytic reactions such as the oxygen evolution/reduction reaction (OER/ORR) [11,12], CO_2_ reduction reaction (CO_2_RR) [13,14], hydrogen evolution reaction (HER) [15,16], and nitrogen reduction reaction (NRR) [17]. Up to now, a series of non-noble-metal SACs, such as Fe, Mn, W, and Mo [18,19,20,21,22], have been proven experimentally and theoretically to possess high catalytic activity for converting N_2_ into NH_3_. More specifically, Mo SACs stand out from these systems. For instance, Li et al. [23] used a single Mo atom anchored on graphene-like 2D gallium nitride (g-GaN) to show excellent catalytic activity with a low overpotential of 0.42 V. Huang et al. [24] theoretically reported efficient and stable electrocatalysts for N_2_ reduction by embedding a single Mo atom on a defective BCN monolayer. Xue et al. [25] found that a single Mo supported on the C_9_N_4_ monolayer exhibits a favorable limiting potential of −0.40 V, which can facilely catalyze N_2_ reduction.

Nevertheless, a main weak point of SACs is that metal atoms tend to migrate on the substrate and further aggregate into metal clusters. To overcome this, identifying the appropriate substrate materials to anchor metal atoms and improving catalytic performance are extremely important. With the advantage of a single-atom active sites distribution, various novel two-dimensional materials, such as graphene [26], h-BN [27], graphitic carbon nitrides [28], MoS_2_ [29], and so on, have been revealed to have immense potential as ideal supporting materials to stabilize metal atoms. Among these, several graphitic carbon nitride platforms, including g-CN [30], g-C_3_N_4_ [31], g-C_2_N [32], and g-C_10_N_3_ [33], have caused widespread concern by virtue of their unique electronic structure and uniformly distributed pores. In addition to the aforementioned graphitic carbon nitrides, Kroke et al. [34] proposed a new hypothetical two-dimensional CN material in 2013, whose unit cell is composed of one C_6_N_7_ motif and one C_3_N_3_ motif connected via C–C bonds, resulting in a graphene-like carbon nitride with a C: N ratio of 9:10 (referred to as g-C_9_N_10_). Li et al. [35] found that g-C_9_N_10_ is stable through the calculations of the phonon spectrum and ab initio molecule dynamics simulation. Xia et al. [36] first theoretically discovered that g-C_9_N_10_ possesses multiple uniformly distributed large pores with abundant sp^2^ N atoms, appealing band structure, as well as excellent thermal stability. Subsequently, it was successfully fabricated through a simple solvothermal method by using heptazine chloride (C_6_N_7_Cl_3_) and cyanuric chloride (C_3_N_3_Cl_3_), which serve as precursors to react with Na in experiments. Combing these remarkable advantages, g-C_9_N_10_, as a novel member of the 2D carbon nitride family, has been successfully applied in the field of electrochemical N_2_ reduction, leading to such compounds as B@g-C_9_N_10_ [36] and V/g-C_9_N_10_ [37]. Inspired by the above-mentioned studies, we speculate that an isolated Mo atom embedded on a g-C_9_N_10_ substrate (Mo@g-C_9_N_10_) would exhibit potential electrocatalytic activity for N_2_ reduction. Herein, in the current work, a comprehensive density functional theory calculation was conducted to evaluate the catalytic activity of Mo@g-C_9_N_10_ toward NRR.

## 2. Results and Discussion

**Figure 1:**
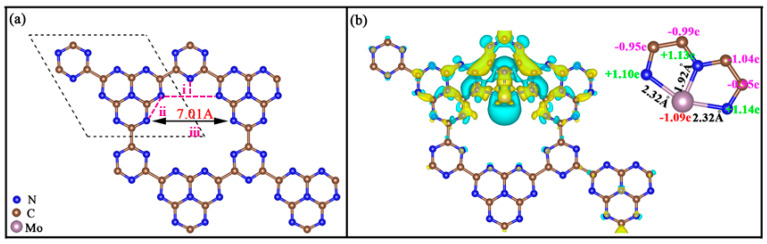
(**a**) Three possible anchoring sites for Mo atom on g-C_9_N_10_ monolayer. (**b**) The charge density difference of Mo@g-C_9_N_10_, where blue and yellow denote the electron density depletion and accumulation area, respectively. The isosurface level is set to 0.003 e/Å^3^.

According to a previous study, replacing the bridging N atom from ideal g-C_3_N_4_ with planar triazine (g-C_3_N_3_) can ultimately obtain the original configuration of g-C_9_N_10_. The symmetry of g-C_9_N_10_ is the same as g-C_3_N_4_ and g-CN, belonging to the P$\bar{6}$m2 space group, but the lattice parameter of g-C_9_N_10_ (9.42 Å) is greater than that of g-C_3_N_4_ (7.13 Å) and g-CN (7.12 Å). In addition, the unit cell of g-C_9_N_10_ involves one heptazine (C_6_N_7_) and one triazine (C_3_N_3_), which are connected by the C-C bond. With the aid of the primitive cell of g-C_9_N_10_, a 2 × 2 × 1 supercell was constructed to anchor the active Mo atom, realized by Mo@g-C_9_N_10_, with the lattice parameter of a = b = 18.83 Å, which is in line with previous DFT result [34]. As depicted in Figure 1a, the g-C_9_N_10_ substrate possesses uniformly distributed pores and a desirable N environment in its atomic motif, offering abundant and uniform N coordinators with lone pair electrons to capture the transition metal ions of ligands. In the present work, the diameter of the cavity is defined as the maximum distance between the N atoms in two embedding sites. Compared to g-C_3_N_4_ (4.13 Å) and g-CN (4.73 Å), g-C_9_N_10_ exhibits a much larger void diameter of 7.01 Å, which makes it a very promising platform for anchoring active atoms.

The structural stability of catalysts takes up a pretty important role in the long-lasting catalytic activity and the likelihood of experimental synthesis, and embedding active atoms will directly induce the alteration of the electronic structure of the catalyst substrate, especially for SACs. In that sense, the strong interaction between a single TM atom and a catalyst substrate is a necessary prerequisite to prevent the diffusion and aggregation of a transition metal atom. Accordingly, the stability of a single Mo atom anchored on a g-C_9_N_10_ monolayer was first estimated by computing the binding energy (*E_b_*) as follows: $E_{b}=E_{Mo@{g-C}_{9}N_{10}}-E_{g-C_{9}N_{10}}-E_{Mo}$, with $E_{Mo@{g-C}_{9}N_{10}}$,$\;E_{g-C_{9}N_{10}}$, and $E_{Mo}$ being the total energies of Mo@g-C_9_N_10_, the pristine g-C_9_N_10_ monolayer, and the isolated Mo atom, respectively. The larger the negative value of $E_{b}$, the better the configuration stability of Mo@g-C_9_N_10_.

As shown in Figure 1a, three possible anchoring sites marked as i, ii, and iii were considered for the Mo atom embedded in the g-C_9_N_10_ monolayer to construct SACs: (i) The Mo atom bonds to three N atoms; (ii) The Mo atom is connected by two adjacent N atoms; (iii) The Mo atom is anchored to the center of the hole. The calculated results indicate that the Mo atom at site iii would ultimately migrate to site i after geometry optimization, and the energetically most favorable location for the Mo atom is site i, with the most negative value of *E_b_* being −7.05 eV. This shows that the Mo atom prefers to bind g-C_9_N_10_ substrate via Mo-N configurations with the corresponding bond lengths of 1.92, 2.32, and 2.32 Å. Such a negative value of E_b_ strongly confirms that the anchored Mo atom would have no inclination to aggregate into clusters. Furthermore, Mo@g-C_9_N_10_ remains a planar configuration after the embedment of the Mo atom on the g-C_9_N_10_ monolayer, further corroborating the good structural stability of Mo@g-C_9_N_10_ configuration. To deepen our insight into the origin of intense binding between Mo atom and g-C_9_N_10_ monolayer, we calculated the charge density difference and the Bader charge, as shown in Figure 1b. It is clear that a remarkable charge redistribution can be found between the anchored Mo atom and the g-C_9_N_10_ monolayer. The result of the Bader charge analysis shows that the active Mo atom transfers about 1.09 electron to pristine g-C_9_N_10_ substrate, ensuring the good thermodynamic stability of Mo@g-C_9_N_10_ SACs.

**Figure 2:**
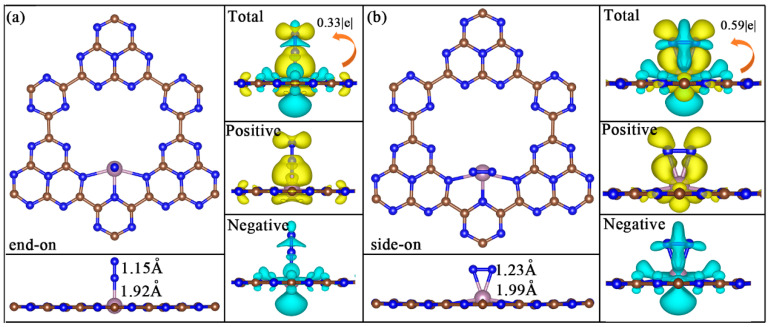
Optimized configurations of N_2_ adsorption with (**a**) end-on and (**b**) side-on patterns on Mo@g-C_9_N_10_ and their corresponding charge density difference, where yellow and cyan represent the positive and negative charges, respectively.

The adsorption and activation of N_2_ molecules on the catalyst surface play a significant role in the N_2_ reduction process, which is an indispensable prerequisite for the follow-up hydrogenation steps. The left side of Figure 2a,b plots the well-optimized N_2_ adsorption configurations on Mo@g-C_9_N_10_ with end-on and side-on patterns, respectively. The Mo-N bond lengths of 1.92 and 1.99 Å for end-on and side-on modes were first noticed, respectively. Beyond that, the adsorption energy of end-on (−1.42 eV) and side-on patterns (−1.37 eV) is negative, showing that Mo SACs exhibit an excellent N_2_ adsorption capability and N_2_ molecules going from the free gas phase to the chemisorbed state is a thermodynamically spontaneous process. In addition, it can be clearly seen that for both adsorption configurations, the N-N bond length was elongated with respect to the free N_2_ molecule (1.12 Å), suggesting the effective activation of the N_2_ molecule. By comparing, the side-on pattern can prominently active the inert N≡N, in which two N atoms of *N_2_ bind to the active Mo atom, leading to a more obvious N-N elongation (1.23 Å), but at the cost of weakening its binding strength with the active Mo atom.

Moreover, the charge density differences for end-on and side-on N_2_ adsorption configurations were further calculated to better understand N_2_ activation, as presented on the right side of Figure 2a,b, respectively. The significant charge redistribution between the embedded Mo atom and N_2_ molecule was observed for both adsorption patterns. The Bader charge analysis reveals that about 0.33 e^−^ and 0.59 e^−^ are fed back to N_2_ with end-on and side-on patterns, respectively, signifying that side-on mode exhibits the stronger N_2_ activation ability.

**Figure 3:**
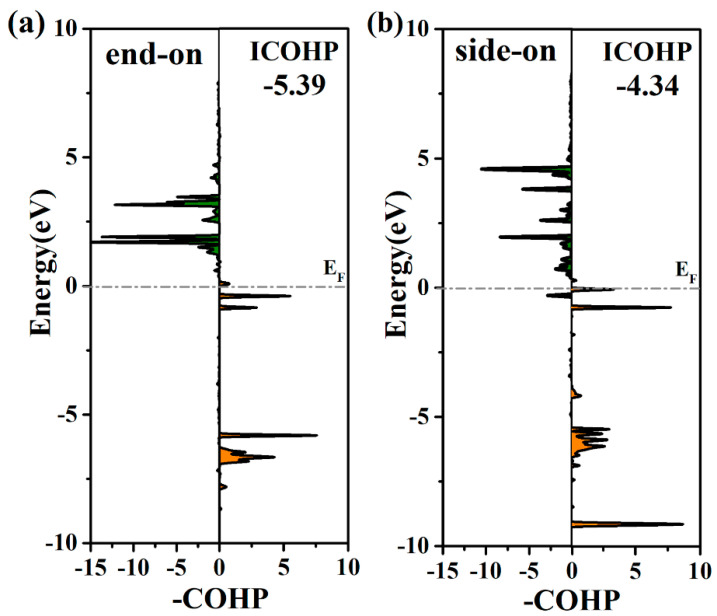
Projected crystal orbital Hamilton population (pCOHP) between Mo and the N atoms of the adsorbed N_2_ via (**a**) end-on and (**b**) side-on configurations, where the right and left parts represent bonding and antibonding contributions, respectively.

To evaluate the Mo-N binding interaction on the surface of Mo@g-C_9_N_10_, we analyzed the projected crystal orbital Hamilton populations (pCOHP), as shown in Figure 3. It is worth noting that the filling of the antibonding orbital population of the side-on pattern increases as compared with that of the end-on pattern, which is well in accordance with the weaker adsorption strength of the N_2_ molecule via side-on configuration. In addition, the integrated-crystal orbital Hamilton population (ICOHP) was performed by integrating the band states up to the highest occupied energy level to quantitatively describe the interaction strength between the active Mo atom and *N_2_ molecule. In general, a more negative value of ICOHP signifies a stronger bonding interaction. The results reveal that the Mo-N binding interaction with the end-on N_2_ adsorption configuration is stronger than with the side-on pattern on the Mo@g-C_9_N_10_ monolayer due to the more negative ICOHP value (−5.39 eV for the end-on configuration, −4.34 eV for the side-on configuration), which agrees well with the above-calculated data. In addition, the details of the COHP analysis of the N–N bond are shown in Figure S1.

**Figure 4:**
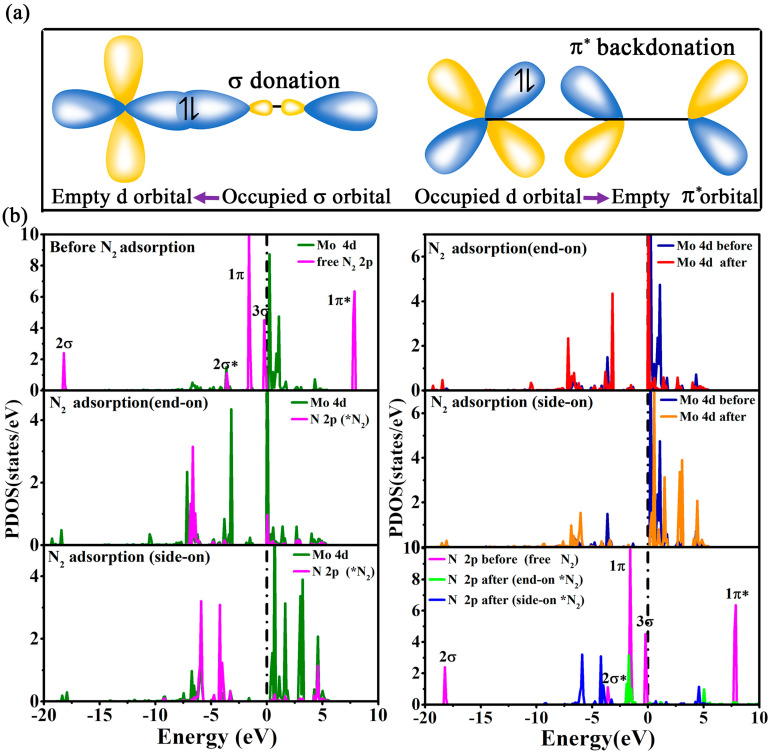
(**a**) Simplified schematic mechanism of nitrogen bonding and activation on transition metal. (**b**) The calculated partial density of states (PDOS) of Mo-4d and N-2p orbitals before and after N_2_ adsorption on Mo@g-C_9_N_10_. *N_2_ represents N_2_ molecule chemisorbed to the substrate binding site.

The activation of N_2_ is usually manipulated by the so-called “push–pull” hypothesis [38]. To be specific, the partially filled d orbitals of the Mo atom can push electrons to the antibonding orbitals of N_2_, thus activating the inert N≡N bonds, and meanwhile, the unfilled d orbitals of the Mo atom can pull the lone-pair electrons from N_2_ to strengthen the Mo-N bond, as revealed in Figure 4a. This mechanism can also be well explained by the partial density of states (PDOS) in Figure 4b, which plots the PDOS for free N_2_ molecule as well as Mo@g-C_9_N_10_ before and after N_2_ adsorption. In detail, the frontier orbitals of an isolated N_2_ molecule possess 2σ, 2σ*, 1π, 3σ, 1π* near the Fermi level, and the Mo-4d orbitals are continuously distributed near the Fermi level before N_2_ adsorption. Upon N_2_ adsorption, the N-2p orbitals are obviously hybridized with the Mo-4d orbitals near the Fermi-level, reflecting that the acceptance of electrons from N_2_ molecule into the empty d orbitals of Mo strengthens N_2_ adsorption to form the Mo-N bonding states. On the other hand, the energy level of antibonding orbitals of N_2_ exhibits a significant down shift as compared with that of free N_2,_ which indicates that some electrons of the Mo atom have been transferred to the adsorbed N_2_ molecule, accompanying the activation of N_2_. It is thus conclusive that the presence of a strong *d*-π* coupling is beneficial for the adsorption and activation of N_2_ on Mo@g-C_9_N_10_, thereby promoting the following hydrogenation.

**Figure 5:**
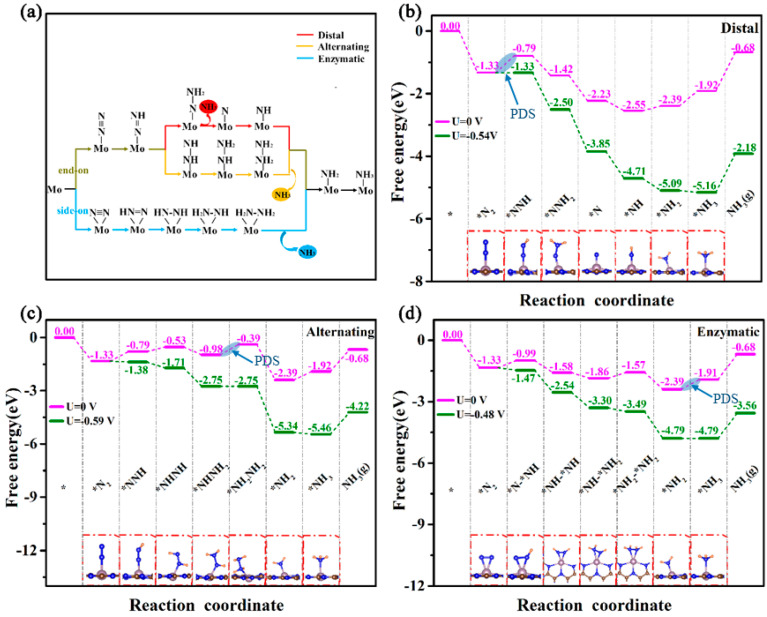
(**a**) Schematic illustration for three reaction pathways for NRR. Free energy diagrams for N_2_ reduction NH_3_ on Mo@g-C_9_N_10_ via (**b**) distal, (**c**) alternating, and (**d**) enzymatic pathway, where inserts represent the fully optimized configurations for reaction intermediates.

To comprehensively evaluate the NRR catalytic activity of Mo@g-C_9_N_10_, the overall reaction mechanisms are examined. In general, the limiting potential ($U_{L}$) is applied as the intrinsic activity criterion for N_2_ reduction conversion into NH_3_, which is obtained by $U_{L}=-∆G_{max}/e$, where $∆G_{max}$ is the maximum free energy among all the hydrogenation steps. As discussed above, the N_2_ molecule can be adsorbed effectively on Mo@g-C_9_N_10_ via end-on and side-on patterns, so we considered three possible reaction pathways, that is, distal, alternating, and enzymatic mechanisms. The initial adsorption configuration and hydrogenation steps for those three reaction pathways are diverse, as exhibited in Figure 5a. The distal and alternating pathways occur by means of N_2_ adsorption with end-on configuration, while the enzymatic mechanism may follow via side-on N_2_ adsorption. In addition, for the distal pathway, the proton–electron pairs (H^+^ + e^−^) first consecutively attack the outermost N atom until the production of the first NH_3_ molecule and then attack the remaining N atom on the surface of Mo@g-C_9_N_10_ catalyst, while for alternating and enzymatic mechanisms, the proton–electron pairs alternatively attack two N atoms of the adsorbed N_2_ molecule.

The NRR Gibbs free energy diagrams for three possible reaction mechanisms are plotted in Figure 5b,c, with the corresponding well-optimized configurations of all the reaction intermediates for each mechanism also provided in the plot. We first examined the NRR performance in the case of the distal mechanism, as shown in Figure 5b. In this mechanism, the terminal N atom of *N_2_ was first attacked by (H^+^ + e^−^) to form *NNH species, with an energy consumption of 0.54 eV. Afterward, the second (H^+^ + e^−^) continuously attacks the distal N atom of *N_2_, resulting in the formation of *NNH_2_ species, and this elementary step becomes downhill by a change of free energy of −0.63 eV. In the third hydrogenation step, the first NH_3_ molecule was released, and only one N remained on the Mo site, with a ∆G value of −0.81 eV. Subsequently, the remaining N atom on the Mo site is gradually protonated into *NH, *NH_2_, and *NH_3_ groups, in which the changes of Gibbs free energy of these three hydrogenation steps are downhill by 0.32 eV, uphill by 0.15 eV and 0.48 eV, respectively. It is clear that, among all the hydrogenation steps, the first hydrogenation step (*N_2_+H^+^ + e^−^ → *NNH) shows the largest increase in Gibbs free energy, which is considered as the potential-determine step (PDS) with a limiting potential of –0.54 V for Mo@g-C_9_N_10_ via the distal mechanism.

When N_2_ reduction follows the alternating mechanism (Figure 5c), we can see that the first elementary step is the same as the step for the distal pathway, accompanied by the formation of *NNH species and the energy input of 0.54 eV. After that, four proton–electron pairs (H^+^ + e^−^) alternately attack the two N atoms of *NNH species to form *NHNH, *NHNH_2_, *NH_2_NH_2_ and *NH_2_ species, producing the first NH_3_ molecule, with the Gibbs free energy changes of 0.26 eV, –0.45 eV, 0.59 eV, and −2.00 eV, respectively, in which the third protonation step (*NHNH_2_+H^+^ + e^−^ → *NH_2_NH_2_) exhibits the largest Gibbs free energy change. Following, the further combination of a (H^+^ + e^−^) with *NH_2_ species generates the second NH_3_ molecule, with an energy injection of 0.48 eV. Note that the PDS of Mo@g-C_9_N_10_ is the formation of *NH_2_NH_2_ species in the alternating route, with the maximum change in the Gibbs free energy of 0.59 eV, which is about 0.05 eV higher than that of the first hydrogenation step.

For the enzymatic mechanism, NRR starts from the side-on N_2_ adsorption configuration, where the proton–electron pairs (H^+^ + e^−^) alternately attack the two N atoms on the active Mo atom, along with the formation of *N-*NH, *NH-*NH, *NH-NH_2_, *NH_2_-NH_2_ intermediates, and the changes in the Gibbs free energy of these elementary steps are 0.34 eV, −0.59 eV, −0.28 eV, and 0.29 eV, respectively. After that, the two N atoms of *NH_2_-NH_2_ species were continuously assaulted by proton–electron pairs (H^+^ + e^−^) to form two NH_3_, in which the production of the second NH_3_ molecule needs an energy of 0.48 eV to drive this process. Hence, the highest energy consumption in the whole NRR process is the last hydrogenation step of *NH_2_ → *NH_3_, which serves as the PDS in the enzymatic mechanism with the limiting potential of −0.48 V. Noticeably, when the applied voltage is −0.48 V, the PDS barrier of *NH_2_ → *NH_3_ can be completely eliminated, meanwhile the other elementary steps have converted into the exothermic process. Overall, N_2_ conversion into NH_3_ on Mo@g-C_9_N_10_ would be more likely to occur via enzymatic mechanisms due to the lower limiting potential compared to other reaction mechanisms.

**Figure 6:**
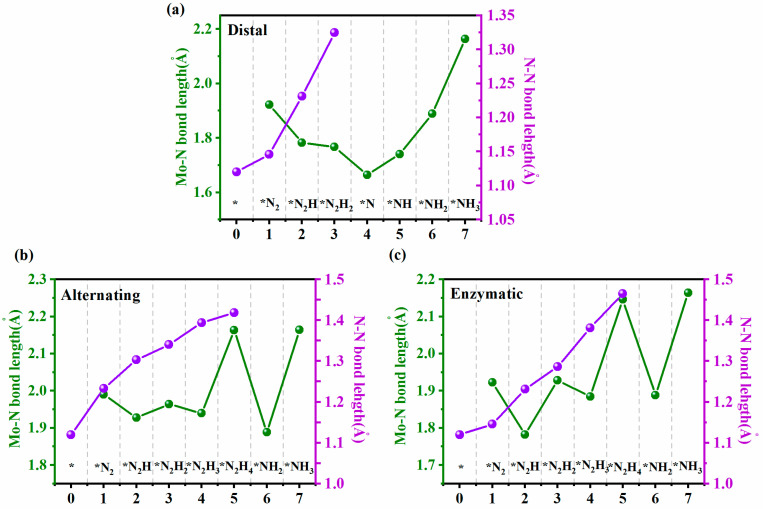
The Mo-N and N-N bond length fluctuation for Mo@g-C_9_N_10_ during N_2_ reduction process through (**a**) distal, (**b**) alternating, and (**c**) enzymatic pathways. The * represents the substrate binding site. The * of intermediates represent species chemisorbed to the substrate binding site.

To elaborate on the origin of N_2_ activation on Mo@-gC_9_N_10_, we investigated the variations of N-N bond length of the adsorbed N_x_H_y_ intermediates as well as the bond length between the active Mo atom and the proximal N atom of N_x_H_y_ intermediates along the three reaction pathways, as illustrated in Figure 6. We first focus our discussions on the changes in the N-N bond length, where before desorbing the first NH_3_ molecule, the N-N bond length is continuously elongated to 1.32 Å, 1.47 Å, and 1.42 Å in the second hydrogenation step of distal, the forth hydrogenation step of alternating and enzymatic reaction pathways, respectively, suggesting the feasibility of N_2_ activation on Mo@g-C_9_N_10_ surface. In addition, the bond length of Mo-N continuously shortens before the production of the first NH_3_ molecule, appearing the shortest Mo-N bond length of 1.66 Å in the third hydrogenation step, and then rapidly elongates as the following hydrogenation progresses, denoting the feasibility of Mo@g-C_9_N_10_ to produce and release the second NH_3_ molecule.

**Figure 7:**
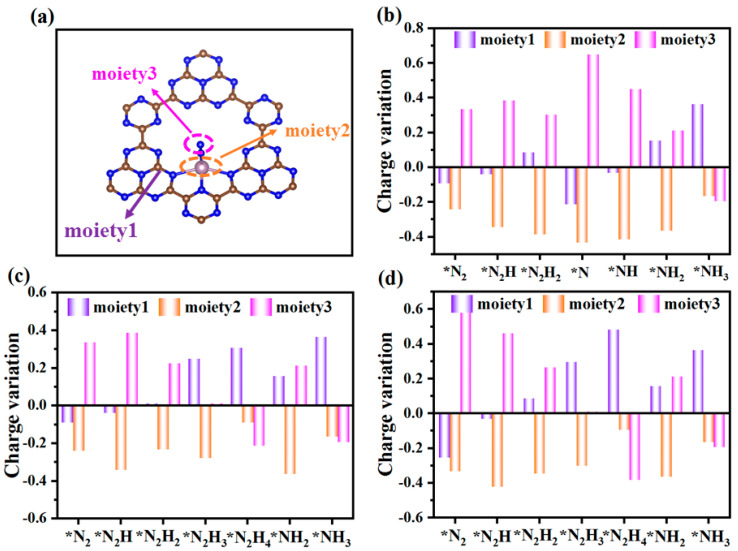
(**a**) Schematic diagram of the definition of three moieties for Mo@g-C_9_N_10_ with the adsorbed NxHy species and their charge variation of three moieties along (**b**) distal, (**c**) alternating, and (**d**) enzymatic pathways. The * of intermediates represent species chemisorbed to the substrate binding site.

To gain more insights into the excellent NRR activity on Mo@g-C_9_N_10_, we have carried out a Bader charge analysis to evaluate the charge variations in each elementary step along three pathways (See Figure 7). According to a previous study [39], each reaction intermediates were here divided into three moieties: the g-C_9_N_10_ monolayer (moiety 1), the anchored Mo atom (moiety 2), and the adsorbed NxHy species (moiety 3), as depicted in Figure 7a. Firstly, the evident charge fluctuations for three moieties along three mechanisms can be observed. What is more, it should be noted that the adsorbed NxHy species can obtain electrons from the g-C_9_N_10_ monolayer and the active Mo atom. The moiety1 serves as an electron reservoir, while mioety2 acts as the communicator for charge transfer between mioety1 and moiety3 throughout the whole N_2_ reduction process, thus contributing to protonation reactions and the excellent catalytic performance for N_2_ fixation.

**Figure 8:**
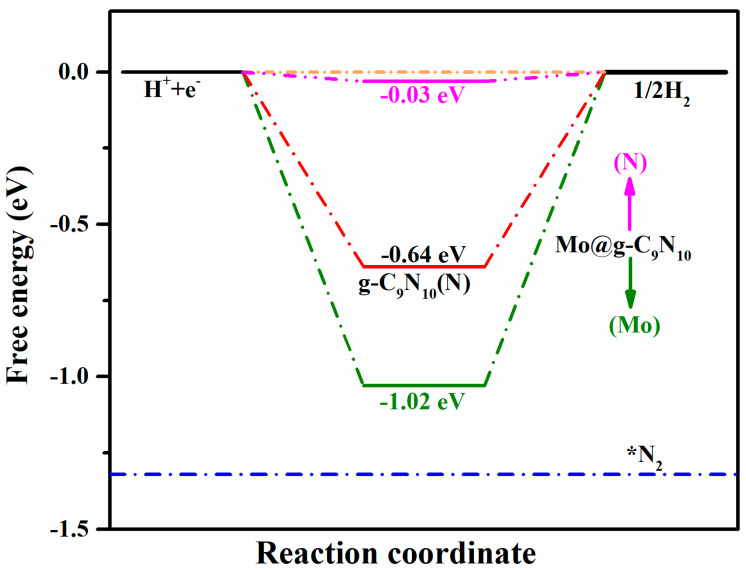
Calculated Gibbs free energy diagram of H atom and N_2_ molecule adsorbed on the Mo@g-C_9_N_10_ matrix. *N_2_ represents N_2_ molecule chemisorbed to the substrate binding site.

An ideal NRR electrocatalyst should possess high catalytic performance and high Faradaic efficiency. Therefore, in addition to the catalytic performance, catalytic selectivity is also a principle factor to consider, in which the HER could consume a certain number of protons and electrons, thereby affecting the Faradaic efficiency of N_2_ reduction. Here, to compare the catalytic selectivity of the Mo@g-C_9_N_10_ candidate, we calculated the Gibbs free energy changes after H and N_2_ adsorption, as provided in Figure 8. It is apparent that the Gibbs free energy of *H at the N site of the pristine g-C_9_N_10_ monolayer is −0.64 eV, which is highly disadvantageous to the HER by hindering the formation of H_2_. However, after embedding a single Mo atom on the g-C_9_N_10_ monolayer, the Gibbs adsorption free energy for the H atom at the N site of the catalyst surface has been raised to −0.03 eV, which is very close to 0, suggesting excellent HER activity. Moreover, compared to the Gibbs adsorption energy of the H atom at the Mo site (−1.02 eV), the N_2_ molecule with side-on configuration exhibits a more negative Gibbs adsorption-free energy at the Mo site (−1.33 eV), which indicates that the active Mo site prefers to occupied by a N_2_ molecule rather than a H atom. Therefore, Mo@g-C_9_N_10_ exhibits high selectivity toward NRR. The calculation results of band structure are given in Supporting Information.

## 3. Computational Details

All the calculations were carried out using the density functional theory (DFT) approach embedded in the Vienna ab initio simulation package (VASP) [40,41]. The projector augmented wave (PAW) method was applied to deal with the ion-electron interactions. The electronic exchange-correlation effect was clarified by the Perdew-Burke-Ernzerhof (PBE) functional within the generalized gradient approximation (GGA) [42]. The kinetic energy cut-off of 500 eV was adopted for the plane-wave expansion of electronic wave function. The Monkhorst-Pack scheme [43] was employed to sample the Brillouin region with a 2 × 2 × 1 k-point grid for structural relaxations, while a 4 × 4 × 1 k-point mesh was applied for electronic structure calculations. The convergence criteria are 0.02 eV∙Å^−1^ and 10^−5^ eV for force and electronic energy, respectively. The empirical density functional dispersion (DFT-D3) correction [44] was used to describe the effect of van der Waals interaction. To minimize the interaction introduced by the periodic units, a vacuum with a thickness of 20 Å along Z directions was inserted. To reveal the charge transfer between active Mo atom and g-C_9_N_10_ substrate, Bader charge analysis was conducted by the quantum theory of atoms in molecules (QTAIM) developed by Professor Richard F.W. Bader and his coworkers [45]. To elucidate the bonding population between atoms, we conducted the projected crystal orbital Hamilton population (pCOHP) analysis, as implemented in the LOBSTER code [46]. Self-consistent iteration of the Kohn Sham equation is used to obtain orbital wave functions of different configurations and the corresponding orbital energy for the calculation of the density of states.

The N_2_ reduction process involves six proton-coupled electron transfer steps ($N_{2}+6H^{+}+6e^{-}\to 2NH_{3}$); each step involves the transfer of one proton combined with an electron from a solution to an adsorbed species on the catalyst surface. Based on the hydrogen electrode model proposed by Nørskov and coworkers [47,48], the chemical potential of the proton–electron pair can be referred to as half that of H_2_ under standard reaction conditions. The Gibbs free energy change for each electrochemical step can be obtained by $∆G=∆E+∆ZPE-T∆S+∆G_{U}+∆G_{pH}$, where ∆E represents the electronic energy difference between reactants and products, ∆ZPE and ∆S denote the changes in zero-point energy and entropy, respectively. The zero-point energy and entropy of reaction species are computed based on the vibrational frequencies, in which only the vibrational modes of adsorption species are considered while the catalyst surface is fixed. $∆G_{U}$ represents the free energy contribution associated with the electrode potential U, which can be obtained by −neU, where n corresponds to the transfer number of electrons. $∆G_{pH}=K_{B}T\times ln10\times pH$ was adopted to consider the free energy correction for pH (in this work, pH = 0).

## 4. Conclusions

In summary, we comprehensively evaluated the potential of Mo@g-C_9_N_10_ as catalysts for electrochemical NH_3_ synthesis by performing DFT calculations. The results indicate that Mo@g-C_9_N_10_ exhibits high thermodynamic stability, excellent catalytic activity, and selectivity. Moreover, the enzymatic pathway is energetically more favorable for Mo@g-C_9_N_10_ to efficiently activate and reduce N_2_ to NH_3_, with a considerable limiting potential of –0.48 V. In particular, the acceptance–donation process is unveiled in combination with the PDOS and the charge density difference of N_2_ adsorbed on Mo@g-C_9_N_10_, illustrating the origin of outstanding catalytic activity for N_2_ reduction. Overall, our work highlights a new family of promising electrocatalysts for N_2_ fixation. We anticipate that this work will provide some meaningful guidance for future theoretical and experimental research on graphitic carbon nitrides-based SACs.

## Data Availability

All data generated or analyzed during this study are included in this published article.

## Supplementary Materials

The following supporting information can be downloaded at: https://www.mdpi.com/article/10.3390/molecules29194768/s1, Figure S1: the crystal orbital Hamil-ton population (COHP) analysis of the N–N bond for N_2_ molecule; Figure S2: Band structures of g-C_9_N_10_ and Mo@g-C_9_N_10_.
