# Supplementary material for: Designing C9N10 Anchored Single Mo Atom as an Efficient Electrocatalyst for Nitrogen Fixation
Source: Molecules. 2024 Oct 9;29(19):4768. doi: 10.3390/molecules29194768 (PMC11478105; doi:10.3390/molecules29194768)
Supplement: Supplementary file 1 [file molecules-29-04768-s001.zip › molecules-3219880-SI.pdf]

Supplementary Information for

Designing C_9N_{10} anchored single Mo atom as an efficient electrocatalyst for nitrogen fixation

Yibo Chen [1], Liang Chen [1], Xinyu Zhang [2], Pengyue Zhang [1,*]

[1] *Hebei College of Industry and Technology, Shijiazhuang 050091, Hebei, China*

[2]*State Key Laboratory of Metastable Materials Science and Technology, Yanshan University, Qinhuangdao 066004, Hebei, China*

*Corresponding Author. zhangpengyueedu@126.com

To gain deep insights into N-N bonding characteristics, the crystal orbital Hamil-ton population (COHP) analysis of the N–N bond was performed, as shown in Fig.S1. In general, the integrated-COHP (ICOHP) can be employed as a measurement of activation degree for N_2 molecule. The less negative the ICOHP, the weaker the N-N bonding, and thus, the more activated is the N_2 molecule. The result shows that the order of ICOHP values are free N_2 molecule (-22.99) < end-on pattern (-21.22) < side-on pattern (-16.44), implying that side-on con figuration can more significantly activates N_2 molecule than end-on con figuration, which is agreement with the analysis of Bader charge.

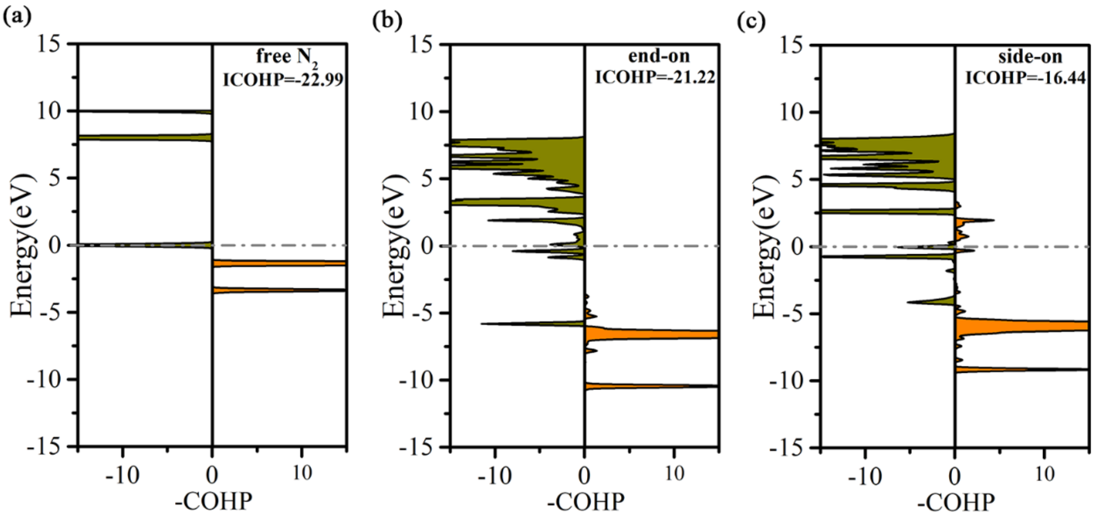

Figure S1. The crystal orbital Hamilton population of N-N bond for (a) the free N_2 molecule, the adsorbed N_2 intermediate via (b) end-on pattern and (c) side-on pattern, respectively.

Besides, the band structure was calculated to provide a physical insight into the high catalytic activity of Mo SACs, as shown in Fig.S2. We found that the band gap of pristine g-C_9N_{10} is 1.52 eV calculated by PBE functions, which matches with the previous study very well[37]. After decorating Mo atom, some impurity states of 4d orbitals were introduced, which greatly increases electronic conductivity and promotes electron transport efficiency in the process of electrochemical N_2 reduction reactions. Overall, good electronic conductivity and high charge transport endow Mo SACs with excellent performance for electrocatalytic N_2 reduction.

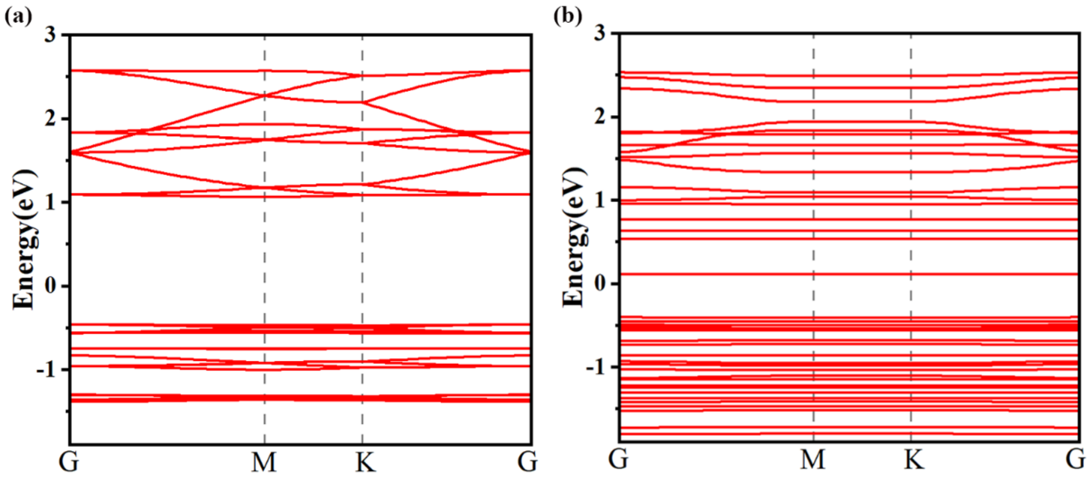

Figure S2. Band structures of (a)g-C_9N_{10} and (b) Mo@g-C_9N_{10}, respectively.
